# A Case of Cataract

**Published:** 1802-08-01

**Authors:** John Morgan

**Affiliations:** Surgeon, of Ipswich


### *A Case of Cataract; communicated by Mr.* JOHN MORGAN, *Surgeon, of Ipswich.*

THE termination of Mr. Crowfoot's cafe of Cataract by inflammation, mentioned in your Journal of May laft, induces me to relate a cafe that occurred within the laft two months. Thomas Damont, aged 52, a patient of my friend Mr. Mat. More, of Bucklefham, in this neighbourhood, made application with two Cataracts. Upon examining the iris of both, which I found to poffefs their natural powers, preference was given to the left eye as being moft convenient for operation. Depreffion was had recourfe to. After paffing the needle in the ufual way, I preffed the lens as low down in the vitreous humour as poffible, in which I fucceeded moft completely; but notwithftanding our fuccefs, we had the mortification to obferve it emerging from the lower part of the iris on the feventh day after the operation.

On the ninth day, an active and extenfive inflammation of the conjunctiva fucceeded, attended with confiderable pain in the fore part of the head, which was, however, without much difficulty removed by the common means. On the fourteenth day, the lens difappeared altogether, and vifion in that eye (allowing for the lofs of a refracting power) is at this time nearly as perfect as ever.

I cannot conclude without commending Mr. Crowfoot for his juft compliment to Mr. Ware's authority; and I truft that the labours of that gentleman, with the communications of his brethren from time to time, will afford further information on the cure of thofe difeafes of the eye to which unfortunately it is fo frequently fubject.

